# Surface Plasmon Resonance Immunosensor with Antibody-Functionalized Magnetoplasmonic Nanoparticles for Ultrasensitive Quantification of the CD5 Biomarker

**DOI:** 10.1021/acsami.2c02936

**Published:** 2022-05-02

**Authors:** Asta Kausaite-Minkstimiene, Anton Popov, Almira Ramanaviciene

**Affiliations:** † Nanotechnas − Center of Nanotechnology and Materials Science, Institute of Chemistry, Faculty of Chemistry and Geosciences, Vilnius University, Naugarduko street 24, LT-03225 Vilnius, Lithuania; ‡ Department of Immunology, State Research Institute Centre for Innovative Medicine, Santariskiu street 5, LT-08406 Vilnius, Lithuania

**Keywords:** surface plasmon resonance, immunosensor, CD5, gold-coated magnetic nanoparticles, signal enhancement

## Abstract

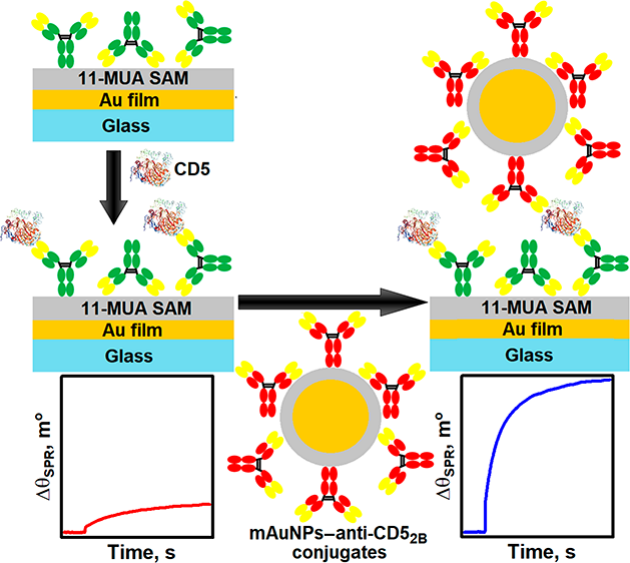

A surface plasmon resonance (SPR) immunosensor signal amplification strategy based on antibody-functionalized gold-coated magnetic nanoparticles (mAuNPs) was developed for ultrasensitive and quantitative detection of the CD5 biomarker using an indirect sandwich immunoassay format. The gold surface of the SPR sensor disk and mAuNPs was modified with a self-assembled monolayer of 11-mercaptoundecanoic acid (11-MUA), and the coupling method using *N*-(3-(dimethylamino)propyl)-*N*′-ethylcarbodiimide hydrochloride and *N*-hydroxysuccinimide was used to immobilize capture antibodies against human CD5 (anti-CD5_2A_) and detection antibodies against human CD5 (anti-CD5_2B_), respectively. The mAuNPs and anti-CD5_2B_ conjugates (mAuNPs–anti-CD5_2B_) were separated by an external magnetic field and used to amplify the SPR signal after the formation of the anti-CD5_2A_/CD5 immune complex on the SPR sensor disk. Compared to the direct CD5 detection with a limit of detection (LOD) of 1.04 nM and a limit of quantification (LOQ) of 3.47 nM, the proposed sandwich immunoassay utilizing mAuNPs–anti-CD5_2B_ significantly improved the LOD up to 8.31 fM and the LOQ up to 27.70 fM. In addition, it showed satisfactory performance in human blood serum (recovery of 1.04 pM CD5 was 109.62%). These results suggest that the proposed signal amplification strategy has superior properties and offers the potential to significantly increase the sensitivity of the analysis.

## 1 Introduction

According to the International Agency for Research on Cancer, oncological diseases are the largest cause of premature death in the world. Nearly 20 million new cases were diagnosed in 2020, with nearly 10 million deaths.^1^ In order to reduce mortality and the cost of treatment and rehabilitation of oncological patients, reliable methods are needed to diagnose the disease at an early stage and to select the most optimal treatment method for each patient. The earlier cancer is diagnosed, the more likely it is that treatment will be effective and patients will have a better quality of life than those diagnosed later.^2^ Thus, the quantification of cancer biomarkers is very important, and the development of new methods with high sensitivity and precision, which would enable the detection of oncological disease at an early stage, is still needed. In recent years, among various bioanalytical methods, surface plasmon resonance (SPR) immunosensors have received great interest and have become one of the most promising methods to detect and quantify a variety of important biomarkers, including biomarkers for cancer.^3^ An important advantage of SPR immunosensors compared to traditional antibody–antigen interaction assay methods is that they are able to provide real-time detection of analytes without any labels. SPR immunosensors are very sensitive, allowing for the detection of extremely low concentrations in a relatively small sample volume. Furthermore, they are able to detect and quantify analytes in solutions with high levels of foreign substances, eliminating the need for a time-consuming sample preparation procedure. The duration of an analysis with the SPR immunosensor often does not exceed a few or several minutes, and multiple analysis can be performed after proper regeneration of the SPR sensor surface.^4^

The suitability of SPR immunosensors for the detection of cancer biomarkers has been demonstrated both in the direct label-free detection format and in various indirect detection formats. Mohseni et al. fabricated a label-free SPR immunosensor for the detection of human matrix metalloproteinase-9 in saliva samples.^5^ The developed immunosensor had a linear range of 10–200 ng/mL with a limit of detection (LOD) of 8 pg/mL, which was lower than most of the other techniques proposed previously. Jena et al. proposed an SPR immunosensor for label-free detection of a Baculoviral inhibitor of the apoptosis repeat containing-5 protein biomarker in serum as low as 6.25 pg/mL.^6^ Although SPR immunosensors can quantify analytes without any labels, low-molecular-weight analytes or analytes at very low concentrations in samples are difficult to detect in direct detection format. In the above-mentioned cases, the interaction between the immobilized ligand and the analyte causes only a small change in the refractive index of the medium resulting in a very small analytical signal. Therefore, the direct format is often not suitable for the detection of nanomolar or lower concentrations. In recent years, this problem has been largely addressed through the use of a variety of nanomaterials. Due to their high mass, these materials provide a much greater change in refractive index, enhance the analytical signal, and increase the sensitivity of the SPR immunosensor. For example, Wang et al. used conjugates of detection antibodies and quantum dots to amplify the analytical signal in a sandwich immunoassay format.^7^ The developed immunosensor made it possible to quantify α-fetoprotein (AFP), carcinoembryonic antigen (CEA), and the cytokeratin 21-1 fragment in clinical samples over a wide concentration range from 1 to 1000 ng/mL with a LOD of 0.1 ng/mL. Ermini et al. proposed an SPR immunosensor for the detection of CEA in blood plasma, the analytical signal of which was amplified using detection antibodies conjugated to gold nanoparticles (AuNPs).^8^ Eletxigerra et al. used streptavidin decorated AuNPs and a dual sandwich amplification strategy to develop an SPR immunosensor for the breast cancer biomarker Epidermal Growth Receptor Factor 2 detection in human serum samples and raw cancer cell lysates.^9^ The LOD for 50% diluted human serum samples was found to be 180 pg/mL. This concentration is 83 times lower than the clinical limit. Krishnan et al. used conjugates of detection antibodies and superparamagnetic particles to detect prostate specific antigen in serum with an ultralow LOD of 10 fg/mL.^10^

Among various nanomaterials, magnetic core–shell nanoparticles consisting of the magnetic nanoparticle and a gold shell (mAuNPs) are highly attractive due to their special properties. mAuNPs not only provide stable binding sites for the immobilization of the biomaterial on their surfaces but also facilitate the separation and concentration of the resulting bioconjugates.^11^ In addition, mAuNPs increase the SPR signal not only due to high mass but also due to electromagnetic enhancement in the evanescent field at the SPR sensor surface caused by the localized surface plasmons excited in the nanoparticles.^12^ However, only a limited number of studies have used mAuNPs to enhance the analytical signal of SPR immunosensors. For instance, a highly sensitive mAuNPs-based SPR immunosensor for the detection of *Mycobacterium tuberculosis* antigen CFP-10 protein was developed by Zou et al.^13^ A sandwich immunoassay was constructed by immobilizing capture anti-CFP-10 antibodies (Ab1) on the surface of the SPR sensor. After immunoreaction of the immobilized Ab1 and CFP-10 protein, mAuNPs functionalized with anti-CFP-10 detection antibodies were used to amplify the SPR signal and increased it 30 times at a LOD of 0.1 ng/mL. The analytical signal of the designed SPR immunosensor depends on the concentration of CFP-10 in the range 0.1–100 ng/mL. Liang et al. used mAuNPs to develop an SPR immunosensor for AFP detection.^14^ Amplification of the analytical signal by the sandwich detection format allowed AFP detection in the concentration range 1.0–200.0 ng/mL with a LOD of 0.65 ng/mL.

CD5, also known as lymphocyte antigen T1, Leu-1, and Ly-1, is a 67 kDa single chain transmembrane glycoprotein expressed by most T-cells, a subset of immunoglobulin M secreting B-cells known as B-1a cells,^15^ regulatory B-cells,^16^ or lymphoma cells, predominant in chronic lymphocytic leukemia, small lymphocytic lymphoma, and mantle cell lymphoma.^17^ Several studies have shown that CD5 expression is a potentially prognostic factor of poor outcome in patients with diffuse large B-cell lymphoma.^18,19^ CD5 is present in ∼80% of T-cell acute lymphoblastic leukemia and T-cell lymphoma^20^ and is considered one of the important biomarkers of malignant T-cells.^21^ However, to our knowledge, no SPR-based immunosensor for the quantification of CD5 has been developed. CD5 levels in biological fluids are very low, so it is necessary to develop an immunosensor with a very high sensitivity.

In this work, we provide our findings of an antibody-functionalized magnetoplasmonic nanoparticles-based SPR immunosensor signal amplification strategy for ultrasensitive quantification of the CD5 biomarker. Capture antibodies against human CD5 (anti-CD5_2A_) were immobilized on the gold surface of the SPR sensor disk coated with an 11-mercaptoundecanoic acid (11-MUA) self-assembled monolayer (SAM). The primary amine groups of the anti-CD5_2A_ interacted with the carboxyl groups of 11-MUA previously activated with a mixture of *N*-(3-(dimethylamino)propyl)-*N*′-ethylcarbodiimide hydrochloride (EDC) and *N*-hydroxysuccinimide (NHS). The gold surface of mAuNPs was modified with 11-MUA SAMs, and detection antibodies against human CD5 (anti-CD5_2B_) were covalently immobilized using the same EDC/NHS coupling chemistry. mAuNPs and anti-CD5_2B_ conjugates (mAuNPs–anti-CD5_2B_) were separated from the colloidal suspension by an external magnetic field. Following immunoreaction of immobilized anti-CD5_2A_ and CD5, mAuNPs–anti-CD5_2B_ conjugates were used to amplify the SPR signal using an indirect sandwich immunoassay format. The proposed signal amplification strategy allowed us to achieve very good sensitivity and to determine femtomolar CD5 concentrations. Furthermore, the ability of the SPR immunosensor to quantify CD5 was tested by analyzing human blood serum with artificially added CD5 and appears to be suitable for analysis of biological samples.

## 2 Experimental Section

### 2.1 Materials and Reagents

Recombinant human CD5 protein (carrier free, predicted molecular mass 39.9 kDa, >95% purity by SDS-PAGE under reducing conditions and visualized by silver stain), anti-CD5_2A_ (monoclonal mouse IgG2A clone #205919, protein A or G purified from hybridoma culture supernatant), and anti-CD5_2B_ (monoclonal mouse IgG2B clone #205910, protein A or G purified from hybridoma culture supernatant) were purchased from R&D Systems (Abingdon, United Kingdom). Sodium dodecyl sulfate (SDS, ACS reagent, ≥99.0% purity, CAS Number: 151-21-3), 11-MUA (98% purity, CAS Number: 71310-21-9), NHS (98% purity, CAS Number: 6066-82-6), EDC (≥98.0% purity, CAS Number: 25952-53-8), 4-(2-hydroxyethyl)-1-piperazineethanesulfonic acid (HEPES, ≥99.5% purity, CAS Number: 7365-45-9), hexane (anhydrous, 95% purity, CAS Number: 110-54-3), methanol (anhydrous, 99.8% purity, CAS Number: 67-56-1), sodium acetate trihydrate (NaAc, ACS reagent, ≥99% purity, CAS Number: 6131-90-4), perchloric acid (HClO_4_, ACS reagent, 70% purity, CAS Number: 7601-90-3), and iron(II) sulfate heptahydrate (FeSO_4_·7H_2_O, ACS reagent, ≥99.0% purity, CAS Number: 7782-63-0) were obtained from Sigma-Aldrich (Steinheim, Germany). Ethanol absolute (≥99.8% purity, CAS Number: 64-17-5) was acquired from Honeywell (North Carolina, USA), refractive index (*n* = 1.518) matching fluid was acquired from Cargille Laboratories (Cedar Grove, New Jersey, USA), hydrogen tetrachloroaurate trihydrate (HAuCl_4_·3H_2_O, ACS reagent, 99.9% purity, CAS Number: 27988-77-8) was acquired from Alfa Aesar (Karlsruhe, Germany), sodium hydroxide (NaOH, pellets, Pharmpur, Ph Eur, BP, NF, CAS Number: 1310-73-2) was acquired from Scharlab S.L. (Sentmenat, Spain), and hydroxylamine hydrochloride (98% purity, CAS Number: 5470-11-1) was acquired from Lach-Ner (Neratovice, Czech Republic). Hexadecyltrimethylammonium bromide (CTAB, ≥99%, for biochemistry, CAS Number: 57-09-0), acetic acid (100%, Ph. Eur., extra pure, CAS Number: 64-19-7), d-sorbitol (≥98%, for biochemistry, CAS Number: 50-70-4), and phosphate buffered saline (PBS, for biochemistry and molecular biology) tablets were received from Carl Roth (Karlsruhe, Germany). Ethanolamine, hydrochloric acid (HCl, fuming, 37% purity, CAS Number: 7647-01-0), and sodium borohydride (NaBH_4_, for analysis, CAS Number: 16940-66-2) were purchased from Merck (Darmstadt, Germany). Iron(III) chloride hexahydrate (FeCl_3_·6H_2_O, pure, CAS Number: 10025-77-1), glycine (for analysis, CAS Number: 56-40-6), and ethylenediaminetetraacetic acid (EDTA, 99% purity, CAS Number: 60-00-4) were obtained from AppliChem (Karlsruhe, Germany). Ultrahigh-quality (UHQ) water was used for the preparation of all aqueous solutions. The 11-MUA solution was prepared in methanol.

### 2.2 Synthesis of mAuNPs

The synthesis of mAuNPs was based on the protocols described by Ramanaviciene et al. and consisted of two steps: the synthesis of Fe_3_O_4_ magnetic nanoparticles and their coating with a gold shell.^22^ The determined average diameter of the synthesized mAuNPs was 47.6 ± 11.3 nm.

### 2.3 Preparation of mAuNPs–anti-CD5_2B_

Prior to conjugation, the mAuNPs’ colloidal suspension was sonicated for 10 min in an ultrasonic bath. Then 1 mL of a colloidal suspension of mAuNPs was added to the test tube. The mAuNPs were collected with a magnet (collection time 10 min), and the supernatant was carefully poured off. The collected mAuNPs were then treated with 500 μL of UHQ water, and the resulting colloidal suspension was sonicated for 1 min. The mAuNPs were again collected with a magnet (collection time 10 min), and the supernatant was poured off. To successfully immobilize antibodies, CTAB used during mAuNP synthesis must be removed from the surface of the nanoparticles.^23^ For this purpose, the mAuNPs were treated with 500 μL of 30 mM NaBH_4_, and the resulting colloidal suspension was sonicated for 1 min and then was stirred with a magnetic stirrer for 1 h. CTAB-free mAuNPs were again collected with a magnet, treated with 500 μL of UHQ water, and sonicated for 1 min. Then solution of 500 μL of 1 mM 11-MUR in methanol was added to the collected mAuNPs. The colloidal suspension was sonicated for 1 min and then stirred for 2 h. After the formation of an 11-MUR SAM on the surface of the mAuNPs, the 11-MUR SAM modified particles were again collected with a magnet and treated with 500 μL of 10 mM PBS, pH 7.4, and the colloidal suspension was sonicated for 1 min. After collecting 11-MUR SAM modified mAuNPs with a magnet and pouring off supernatant, the carboxyl groups of the 11-MUA SAM were activated with a mixture of 200 μL of 0.4 mM EDC and 200 μL of 0.1 mM NHS for 15 min after colloidal solution sonication for 1 min. Finally, after collection of the activated mAuNPs with a magnet, pouring off the supernatant, and washing the particles with 500 μL of 10 mM PBS, pH 7.4, with subsequent collection with a magnet, 500 μL of 133.33 nM anti-CD5_2B_ solution in PBS, pH 7.4, was added and the resulting colloidal suspension was stirred for 2 h. Then the mAuNPs–anti-CD5_2B_ conjugates were collected with a magnet, the supernatant with unbound antibodies was poured off, and the mAuNPs–anti-CD5_2B_ conjugates were washed with 500 μL of 10 mM PBS, pH 7.4, with subsequent collection with a magnet. Then, the mAuNPs–anti-CD5_2B_ conjugates were diluted to 500 μL in 10 mM PBS, pH 7.4, and the resulting colloidal suspension was stirred for 15 min and stored at +4 °C.

### 2.4 SPR Sensor Disk Surface Preparation and Immobilization of anti-CD5_2A_

The surface of the new gold-coated SPR sensor disk (SD AU, XanTec Bioanalytics GmbH, Muenster, Germany) was cleaned by incubation in methanol for 30 min and hexane for 2 min and finally washed with UHQ water. The sensor disk was then immersed in a 1 mM solution of 11-MUA in methanol and kept there for 24 h at room temperature. The 11-MUA SAM modified sensor disk (Au/11-MUA) was washed with methanol and UHQ water, dried in an ambient environment, and placed on a hemicylinder mounted on a slider via a refractive index matching fluid. The slider was installed in a double channel SPR-analyzer Autolab Esprit (Metrohm Autolab BV, Utrecht, The Netherlands), and a cuvette (surface area of 7.9 mm^2^ in one channel) was placed. The Au/11-MUA surface stabilization/rehydration was carried out by incubation for approximately 30 min at 2 min intervals in 10 mM NaAc coupling buffer, pH 4.5, and 10 mM glycine/HCl regeneration solution, pH 2.0, until a stable baseline was obtained. Anti-CD5_2A_ antibodies were immobilized in both channels of the SPR cuvette. The Au/11-MUA surface was first treated with coupling buffer for 200 s. A 1:1 mixture of 0.4 M EDC and 0.1 M NHS in water was then used to activate the 11-MUA carboxyl groups. The duration of activation was 600 s. After activation, the EDC/NHS mixture was removed by rinsing the cuvette with coupling buffer, and the resulting surface with active NHS esters was exposed to 500 nM anti-CD5_2A_ in coupling buffer for 1800 s. This results in robust amide bond formation between the primarily amine groups of anti-CD5_2A_ and the carboxyl groups of 11-MUA (Au/anti-CD5_2A_). After washing the cuvette with coupling buffer and removing unbound anti-CD5_2A_ from the cuvette, the deactivation of the remaining activated carboxyl groups was carried out using 1 M ethanolamine solution, pH 8.5, for 600 s. Finally, Au/anti-CD5_2A_ was incubated in 10 mM PBS running buffer, pH 7.4, and regeneration solution at 2 min intervals for approximately 30 min until a stable baseline was obtained.

### 2.5 Anti-CD5_2A_/CD5 Interaction and Regeneration of the Au/anti-CD5_2A_

After reaching a stable baseline in 10 mM PBS running buffer, pH 7.4, for 200 s, a CD5 solution in running buffer was injected into one channel of the SPR cuvette (measurement channel). The other channel was used as the reference (reference channel). Running buffer without CD5 was injected into the reference channel for the negative control. The affinity interaction between the covalently immobilized anti-CD5_2A_ and CD5 present in the solution was registered for 600 s, and then the dissociation by a running buffer for 200 s was performed. The Au/anti-CD5_2A_ was then regenerated with a 10 mM glycine/HCl regeneration solution, pH 2.0. The regeneration was carried out for 300 s. Finally, the baseline was restored by exposure of Au/anti-CD5_2A_ in 10 mM PBS running buffer, pH 7.4, and, if necessary, a solution with a different concentration of CD5 was analyzed. The difference between the measurement and the reference channels was used to evaluate the anti-CD5_2A_/CD5 interaction.

### 2.6 Signal Amplification Using mAuNPs–anti-CD5_2B_

An indirect sandwich immunoassay format was used for signal amplification. After the formation of the anti-CD5_2A_/CD5 immune complex on a sensor surface (Au/anti-CD5_2A_/CD5), its interaction with detection anti-CD5_2B_ antibodies conjugated to mAuNPs was investigated. After reaching a stable baseline (200 s), Au/anti-CD5_2A_ was incubated in CD5 solution in running buffer in the measurement channel of the cuvette for 600 s, followed by washing with running buffer for 100 s. The colloidal suspension of mAuNPs–anti-CD5_2B_ in 10 mM PBS, pH 7.4, was then injected into the measurement channel, and the interaction between Au/anti-CD5_2A_/CD5 and the conjugates was registered for 600 s, followed by dissociation in a running buffer for 100 s. Au/anti-CD5_2A_ was regenerated with a 10 mM glycine/HCl regeneration solution, pH 2.0, for 300 s, and the baseline was restored by running buffer treatment. The reference channel was filled with the pure running buffer. The difference between the measurement and reference channels was used to evaluate the interaction.

### 2.7 Analysis of CD5 Spiked Human Blood Serum Samples

CD5 spiked human blood serum was used to simulate the analysis of real samples. The 10 times with 10 mM PBS, pH 7.4, diluted serum was used in this experiment. Samples were prepared by using the addition technique according to which the diluted serum was spiked with a known concentration of CD5: 1.04, 26.02, and 63.53 pM. The principle of CD5 detection was the same as described above for standard CD5 samples. First, Au/anti-CD5_2A_ was incubated with serum spiked with CD5, and then, a colloidal suspension of mAuNPs–anti-CD5_2B_ in 10 mM PBS, pH 7.4, was injected into the SPR cuvette measurement channel. Pure running buffer was injected into the reference channel. The difference between the measurement and reference channels was used to evaluate the interaction. The measurement was repeated three times for a serum sample with the same CD5 concentration.

### 2.8 Calculations

The surface concentration of immobilized capture anti-CD5_2A_ antibodies was calculated from a linear relationship between the SPR angle shift and the amount of bound biomolecule. The change in the 120 m° SPR angle corresponds to a change in the biomolecules’ surface concentration of 1 ng/mm^2^. The regeneration efficiency was evaluated based on the baseline SPR signal recorded before Au/anti-CD5_2A_ and CD5 interaction (*θ*_*SPR before interaction*_) and the baseline SPR signal recorded when the Au/anti-CD5_2A_/CD5 was treated with regeneration solution and finally 10 mM PBS, pH 7.4 (*θ*_*SPR after interaction*_):
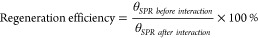


The SPR signal generated interacting anti-CD5_2A_ and CD5 or anti-CD5_2A_/CD5 immune complex and mAuNPs–anti-CD5_2B_ under steady-state conditions (equilibrium angle) was calculated by approximating the results obtained during the association phase according to the hyperbolic function *y* = *ax*/(*b* + *x*), where the parameter *a* is the equilibrium angle, m°.

The LOD was estimated as the concentration of CD5 that gives an SPR signal equal to 3 standard deviations of the baseline noise. Meanwhile, the limit of quantification (LOQ) was estimated as the concentration of CD5 that gives the SPR signal equal to 10 standard deviations of the baseline noise. All experimental results were represented as a mean value of three independent measurements with the error bars.

## 3 Results and Discussion

**Figure 1:**
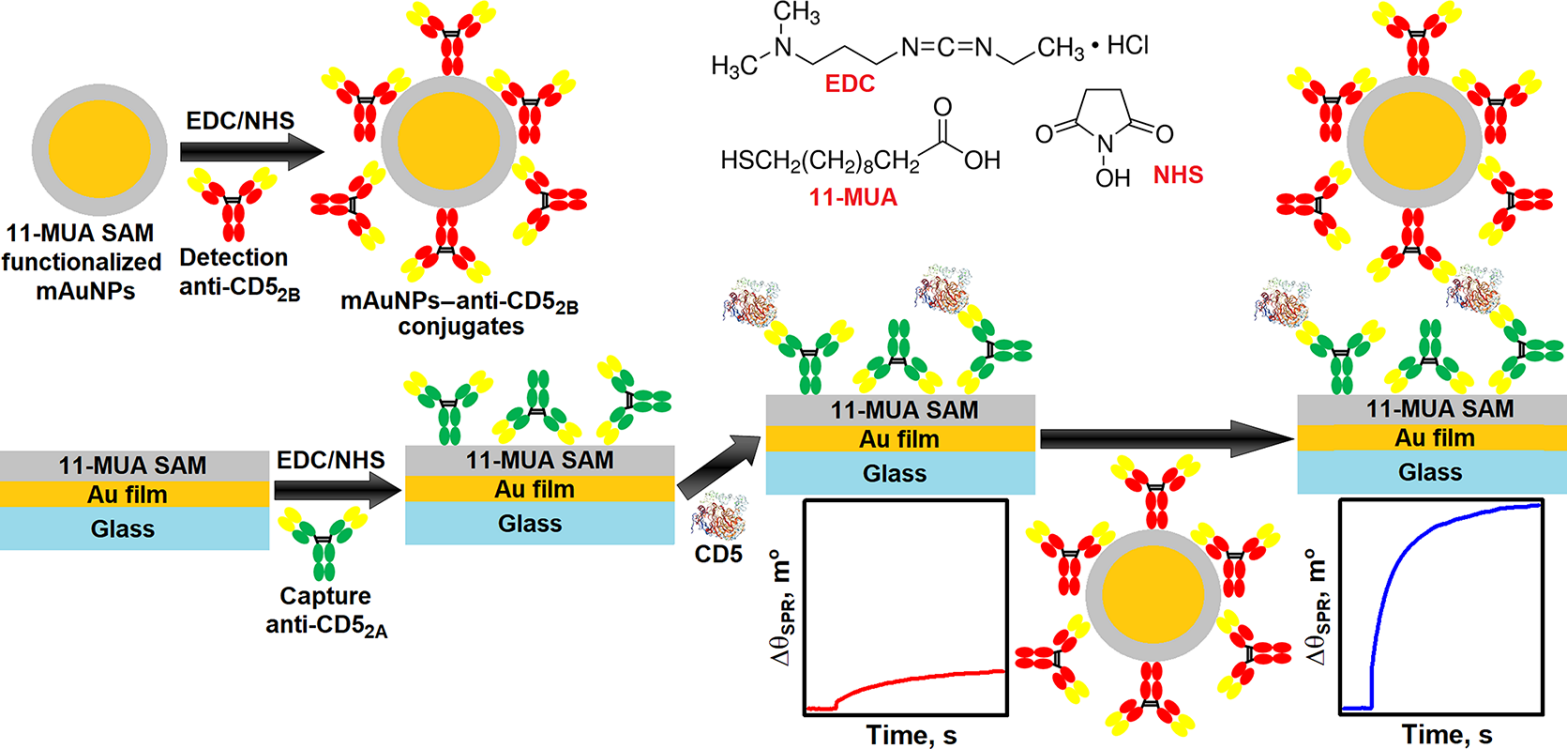
Simplified schematic illustration of mAuNPs–anti-CD5_2B_ conjugate formation and the SPR-based direct and indirect sandwich immunoassay for CD5 detection.

In this study, a signal enhancement strategy for the SPR immunosensor was developed for the quantification of the CD5 biomarker. To achieve this, capture anti-CD5_2A_ antibodies were immobilized on the gold surface of the SPR sensor disk modified with an 11-MUA SAM via their primary amino groups by activating the carboxyl groups of 11-MUA with a mixture of EDC and NHS. The gold surface of the mAuNPs was modified with an 11-MUA SAM, and detection anti-CD5_2B_ antibodies were covalently immobilized using the same EDC/NHS coupling chemistry. mAuNPs–anti-CD5_2B_ conjugates were collected from the colloidal suspension by an external magnetic field. Following the immunoreaction of immobilized anti-CD5_2A_ and CD5 in a solution, mAuNPs–anti-CD5_2B_ conjugates were used to amplify the SPR signal in an indirect sandwich immunoassay format. Since the SPR signal depends on the change in the refractive index of the medium near a metal surface, which is proportional to the molecular weight of the bound analyte, the binding of larger molecules results in a higher signal. Therefore, due to the high mass of mAuNPs–anti-CD5_2B_, the signal of the immunosensor increased significantly and became suitable for the detection of significantly lower concentrations of CD5. The main principles of mAuNPs–anti-CD5_2B_ preparation, immobilization of capture anti-CD5_2A_ antibodies, detection of CD5 by a direct immunoassay format, and amplification of the SPR signal using mAuNPs–anti-CD5_2B_ in an indirect sandwich immunoassay format are shown in Figure 1.

In order to determine the optimal conditions for CD5 detection, the concentration of the capture anti-CD5_2A_ antibody solution used for immobilization and the duration of immobilization were first optimized. A carbodiimide–succinimide immobilization method based on the activation of carboxyl functional groups using a mixture of EDC and NHS in water was chosen for immobilization. Activation of the carboxyl groups available on the SPR sensor surface with this mixture yields highly reactive *N*-hydroxysuccinimide esters that react with the primary amino functional groups of the proteins, forming a strong amide bond between the protein and the surface. Antibodies are immobilized in a random manner due to their asymmetric structure, and some of their antigen-binding sites might be inaccessible to the analyte. Despite this, the EDC/NHS coupling technique is very simple and easy to perform, does not require additional antibody modification, and is therefore frequently used in the development of SPR immunosensors. Solutions of 200.0, 300.0, and 500.0 nM anti-CD5_2A_ in 10 mM NaAc coupling buffer, pH 4.5, were tested to optimize the amount of anti-CD5_2A_ used for immobilization. To increase the ligand coupling yield during immobilization, it is recommended to increase its concentration near the surface of the SPR sensor due to electrostatic interaction. For this, the surface must have a negative charge and the amino group of the antibodies a positive charge. The pI of human IgG2 subclass antibodies is 7.4 ± 0.6.^24^ The p*K*_a_ of a surface-attached acid through the SAM formation is defined as the pH value of the solution that is in contact with the modified surface when half of the SAM acid functional groups are ionized^25^ and depends on many factors, including the nature of the surface-attached acid, as well as the composition and ionic strength of the solution. The p*K*_a_ of an 11-MUA SAM deposited on a gold film in 1.0 M NaClO_4_ solution was determined to be 4.4 ± 0.2. The p*K*_a_ value of the 11-MUA SAM, determined by potentiostatic infrared titration, was 4.3,^26^ while the p*K*_a_ value 3.3 ± 0.1 was detected for 11-MUA at an AuNPs/aqueous interface.^27^ Therefore, taking this into account, when the pH value of the buffer solution is 4.5, anti-CD5_2A_ has a positive charge, while the remaining nonactivated 11-MUR carboxyl functional groups have a negative charge. Electrostatic interaction increases the concentration of anti-CD5_2A_ near the sensor surface, resulting in an increase in the surface concentration of the immobilized antibody, which determines the magnitude of the SPR signal generated by the antibody–analyte interaction and, therefore, the sensitivity of the assay.

**Figure 2:**
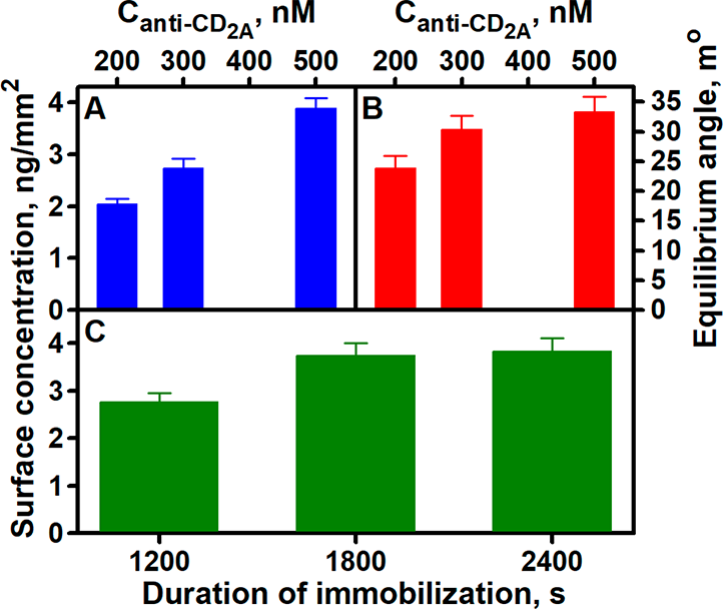
Dependence of the anti-CD5_2A_ surface concentration on (A) the concentration of these antibodies in the solution and (C) the immobilization duration. Impact of the anti-CD5_2A_ surface concentration on the SPR signal (B). Experimental conditions: (A and B) 1800 s duration of immobilization; (B) 50.14 nM CD5; (C) 500.0 nM anti-CD5_2A_.

As shown in Figure 2A, the surface concentration of immobilized anti-CD5_2A_ increased with increasing concentration of its solution: 2.04 ± 0.11 (200.0 nM), 2.73 ± 0.19 (300.0 nM), and 3.81 ± 0.21 (500.0 nM). The calculated SPR signal induced by the interaction of immobilized anti-CD5_2A_ with the same amount of CD5 (50.14 nM) also increased with increasing concentration of antibody solution and was 23.82 ± 2.12, 30.36 ± 2.30, and 33.29 ± 2.60 m° at 200.0, 300.0, and 500.0 nM, respectively (Figure 2B). As can be seen from the results, the use of 500 nM anti-CD5_2A_ results in a surface concentration 1.40 times higher than 300 nM, but the SPR signal induced by the anti-CD5_2A_ and CD5 interaction increases only slightly. Based on the results obtained, it can be stated that a further increase in the anti-CD5_2A_ concentration will not cause a significant increase in the SPR signal during the interaction. Thus, the optimal anti-CD5_2A_ concentration can be considered to be 500.0 nM.

The surface concentration depends not only on the concentration of the antibody solution used for immobilization but also on the duration of immobilization. Figure 2C shows that the surface concentration of anti-CD5_2A_ increases with increasing duration of immobilization. The surface concentration of anti-CD5_2A_ was found to be 2.76 ± 0.19, 3.73 ± 0.28, and 3.82 ± 0.29 ng/mm^2^ at an immobilization duration of 1200, 1800, and 2400 s, respectively. Comparing the obtained results, it can be seen that the increase of the surface concentration after 1800 s becomes insignificant, and the duration of the whole immobilization process is significantly extended. Therefore, 1800 s can be considered as the optimal duration of immobilization.

Regeneration of the surface of the SPR immunosensor allows it to be used for multiple analyses. The goal of regeneration is to disrupt the antibody–antigen immune complex without reducing the activity of the immobilized antibody or affecting its structure. During regeneration, molecules adsorbed on the surface due to nonspecific sorption can also be removed from the immunosensor surface. If the antibody–antigen complex is not disrupted, then the antibody immobilized on the surface of the immunosensor can no longer interact with the analyte, and a lower SPR signal can be recorded at the same concentration. Therefore, to avoid errors in the analysis, it is very important to select a suitable solution for regeneration, which would ideally have a recovery efficiency of 100% or as high as possible. Regeneration solutions having acidic or basic pH as well as surfactants were used to regenerate the Au/anti-CD5_2A_/CD5 surface. After association of immobilized anti-CD5_2A_ with 50.14 nM CD5 in 10 mM PBS, pH 7.4, and subsequent dissociation of the anti-CD5_2A_/CD5 immune complex, the surface of the immunosensor was exposed to the regeneration solution for 300 s, and the regeneration efficiency was evaluated from the recorded sensogram.

**Figure 3:**
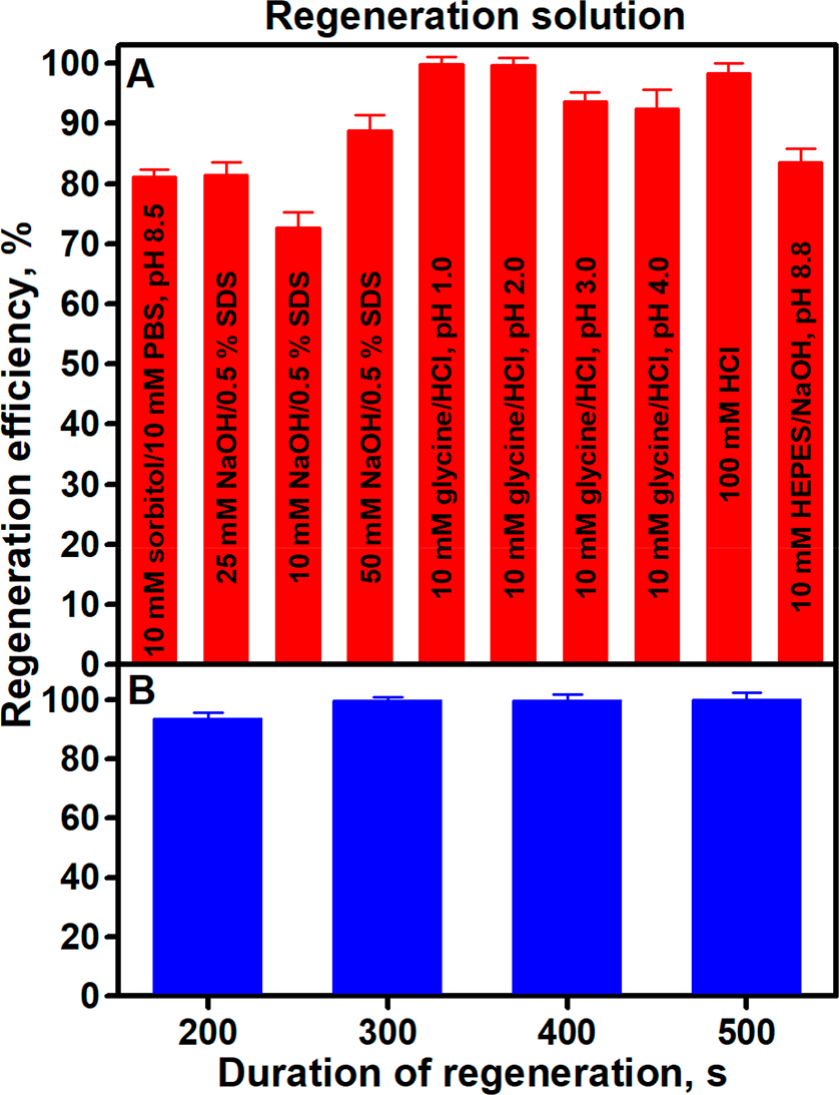
Dependence of the Au/anti-CD5_2A_/CD5 surface regeneration efficiency on (A) the regeneration solution and (B) the duration of regeneration. Experimental conditions: (A) 1800 s duration of 500.0 nM anti-CD5_2A_ immobilization, 50.14 nM CD5, 300 s duration of regeneration; (B) 10 mM glycine/HCl, pH 2.0, regeneration solution.

The experimental results presented in Figure 3A show that the solutions with the best regeneration efficiency were 10 mM glycine/HCl, pH 2.0 (99.77 ± 1.15%), and 10 mM glycine/HCl, pH 1.0 (99.88 ± 1.18%). As can be seen, the regeneration efficiencies of these solutions were very similar, so it was decided to use a less drastic pH solution to avoid possible inactivation of immobilized anti-CD5_2A_ during repeated regeneration. By examining the dependence of the regeneration efficiency of 10 mM glycine/HCl, pH 2.0, on the duration of regeneration (Figure 3B), it was observed that the regeneration efficiency improved with increasing regeneration duration and was 93.52 ± 2.40, 99.77 ± 1.15, 99.87 ± 2.00, and 99.93 ± 2.64% for the regeneration durations of 200, 300, 400, and 500 s, respectively. It is obvious that the regeneration efficiency practically does not improve after 300 s; therefore, due to the possible inactivation of anti-CD5_2A_ during repeated regeneration, the optimal duration of Au/anti-CD5_2A_/CD5 surface regeneration was assumed to be 300 s.

**Figure 4:**
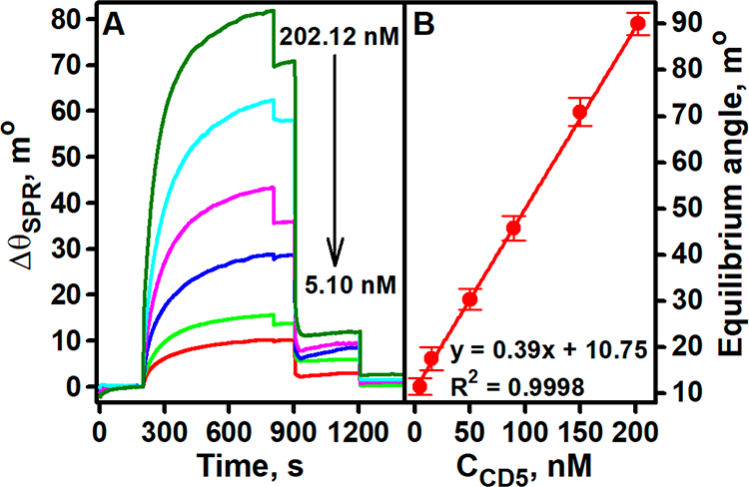
SPR sensograms recorded during analysis of solutions with different CD5 concentrations using (A) a direct immunoassay format and (B) a calibration curve. Experimental conditions: 1800 s duration of 500.0 nM anti-CD5_2A_ immobilization, 300 s duration of regeneration, 10 mM glycine/HCl, pH 2.0, regeneration solution.

The sensitivity of the optimized SPR immunosensor was examined by analyzing solutions of different concentrations of CD5 in 10 mM PBS, pH 7.4. The surface of Au/anti-CD5_2A_ was exposed to 10 mM PBS, pH 7.4, until a stable SPR angle was established, and then, CD5 solution was injected into the SPR cuvette measurement channel. The formation of the anti-CD5_2A_/CD5 immune complex caused an increase in the SPR angle (Figure 4A). PBS buffer was injected at the end of the association phase, resulting in partial dissociation of the resulting immune complex. Finally, the Au/anti-CD5_2A_/CD5 was treated with 10 mM glycine/HCl, pH 2.0, regeneration solution, and then 10 mM PBS, pH 7.4, in order to restore the baseline.

Figure 4A shows the SPR sensograms recorded for solutions with different concentrations of CD5 ranging from 5.10 to 202.12 nM. The experimental results show that the surface of Au/anti-CD5_2A_ was almost completely regenerated. This definitely indicates a well-optimized regeneration procedure. It can also be seen that the shift in the SPR angle was dependent on the concentration of CD5 in the sample. For each sensogram, the SPR signal under steady-state conditions (equilibrium angle) was calculated and plotted against the CD5 concentration (Figure 4B). A linear relationship was observed with *R*^2^ = 0.9998 between CD5 concentrations in the range of concentrations studied. The LOD was estimated to be 1.04 nM and the LOQ 3.47 nM at a signal-to-noise ratio of 3 and 10, respectively.

**Figure 5:**
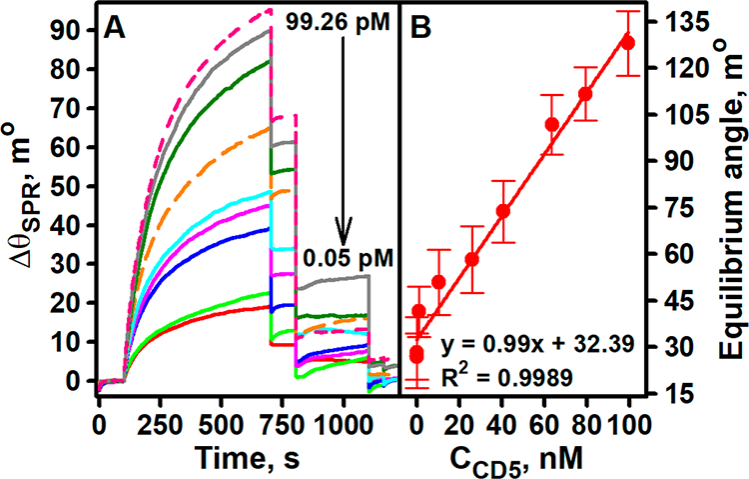
SPR sensograms recorded during the analysis of solutions with different concentrations of CD5 using (A) an indirect sandwich immunoassay format and (B) a calibration curve. Experimental conditions: 1800 s duration of 500.0 nM anti-CD5_2A_ immobilization, 300 s duration of regeneration, 10 mM glycine/HCl, pH 2.0, regeneration solution.

One of the main goals in the development of SPR immunosensors, as well as other biosensors, is the ability to apply them to the testing of biological samples, such as urine, saliva, blood plasma, or serum. Furthermore, matrix effects caused by species of high molecular weight present in any complex biological samples hinder the application of SPR immunosensors. Therefore, such samples should normally be diluted in buffer before analysis.^28^ Because the concentrations of various analytes in biological samples are very low, the immunosensor must be able to detect nanomolar or even femtomolar concentrations in order to be suitable for the analysis of these samples. The levels of soluble CD5 circulating in human serum are relatively very low, ranging from 1 to 24 ng/mL (0.015–0.36 nM). In the serum of healthy subjects, CD5 is detected at concentrations with a median of 1.75 ng/mL^29^ (0.044 nM). Elevated serum CD5 levels have been reported in patients with certain autoimmune diseases, such as Sjogren’s syndrome,^30^ rheumatoid arthritis^31^ or atopic dermatitis,^32^ as well as some non-autoimmune diseases, such as septic syndrome,^33^ bladder cancer,^34^ non-small cell lung cancer,^35^ and others. Therefore, the immunosensor signal that is recorded during the direct anti-CD5_2A_ and CD5 interaction is inefficient to detect such low CD5 levels in biological samples. Thus, an indirect sandwich immunoassay format was used to increase the sensitivity and reduce the detection limit. Since the SPR signal of the immunosensor depends on the change in the refractive index of the medium near the metal surface, thus, the signal was significantly increased due to the high mass of mAuNPs–anti-CD5_2B_. In addition, the SPR signal was increased due to the electromagnetic enhancement in the evanescent field at the SPR sensor surface caused by the localized surface plasmons excited in the nanoparticles. Following anti-CD5_2A_ and CD5 immunoreaction, a colloidal suspension of mAuNPs–anti-CD5_2B_ in 10 mM PBS, pH 7.4, was injected into the measuring channel of the SPR cuvette. The interaction of Au/anti-CD5_2A_/CD5 with conjugates was observed for 600 s, followed by dissociation in PBS buffer for 100 s and regeneration using 10 mM glycine/HCl, pH 2.0, for 300 s. Finally, the baseline was restored by treatment with PBS buffer. Sensograms recorded during the Au/anti-CD5_2A_/CD5 and mAuNPs–anti-CD5_2B_ interaction are shown in Figure 5A.

An increase in SPR signal was registered with increasing CD5 concentration, indicating an effective interaction between Au/anti-CD5_2A_/CD5 and mAuNPs–anti-CD5_2B_. A linear relationship between the CD5 concentration and the calculated equilibrium angle (*R*^2^ = 0.9989) was observed over the entire range of concentrations studied from 0.05 to 99.26 pM (Figure 5B). The LOD was estimated to be 8.31 fM and the LOQ 27.70 fM. Compared to the direct detection format, both LOD and LOQ increased drastically. The detection range of the standard ELISA kit for the analysis of human soluble CD5 is 15.6–1000.0 pg/mL (0.39–25.06 pM). Thus, the signal amplification strategy proposed in this work allows the detection of significantly lower CD5 concentrations. A comparison of the analytical characteristics of the developed SPR immunosensor signal amplification strategy with some reported SPR immunosensors based on signal amplification with high-mass antibody-functionalized nanoparticles is presented in Table 1. As can be seen, the proposed strategy achieves similar or even significantly better analytical characteristics.

**Table 1:** Comparison of Analytical Characteristics of Some SPR Immunosensors Based on Signal Amplification with High-Mass Antibody-Functionalized Nanoparticles^a^

High-mass conjugates	Analyte	LOD	Linear range	Reference
mAuNPs–anti-CD5_2B_	CD5	8.31 fM		This work
QDs–anti-AFP	AFP	0.1 ng/mL		(7)
QDs–anti-CEA	CEA	0.1 ng/mL		
QDs–anti-CYFRA 21-1	CYFRA 21–1	0.1 ng/mL		
AuNPs_str_–anti-CEA_biot_	CEA	0.1 ng/mL		(36)
mAuNPs–anti-AFP	AFP	0.65 ng/mL	1.0–200.0 ng/mL	(14)
mAuNPs–anti-CFP-10	CFP-10	0.1 ng/mL	0.1–100.0 ng/mL	(13)
AuNPs_str_–anti-CEA_biot_	CEA	88.8 fM		(8)
AuNPs–anti-cTnT	cTnT	0.5 ng/mL	0.5–40 ng/mL	(37)
MWCNTs–anti-TauP	TauP	125 pM	125–1000 pM	(38)
AuNPs_str_	ErbB2	180 pg/mL	0.23–55 ng/mL	(9)
MBs_str_	BNP	25 pg/mL		(39)
MBs_str_	SEB		100–1000 pg/mL	(40)
MBs–anti-βhCG	βhCG	0.45 pM		(41)

a QDs, quantum dots; AFP, α-fetoprotein; anti-AFP, antibodies against AFP; CEA, carcinoembryonic antigen; anti-CEA, antibodies against CEA; CYFRA 21-1, cytokeratin fragment 21-1; anti-CYFRA 21-1, antibodies against CYFRA 21-1; AuNPs_str_, streptavidin modified AuNPs; anti-CEA_biot_, biotinylated antibodies against CEA; CFP-10, culture filtrate protein; anti-CFP-10, antibodies against CFP-10; cTnT, cardiac troponin T; anti-cTnT, antibodies against cTnT; MWCNTs, multiwalled carbon nano tubes; TauP, Tau protein; anti-TauP, antibodies against TauP; ErbB2, human epidermal growth factor receptor 2; MBs_str_, streptavidin modified magnetic beads; BNP, brain natriuretic peptide; SEB, Staphylococcal enterotoxin B; βhCG, β human chorionic gonadotropin; anti-βhCG, antibodies against βhCG.

The sensitivity and specificity of the developed indirect sandwich format SPR immunosensor were assessed by testing human serum samples. A series of samples were prepared by spiking 10-fold diluted serum with CD5 at different concentrations. Each serum sample was tested in triplicate. Sensograms were recorded, and the equilibrium angles and their average values for each CD5 concentration were calculated. The CD5 concentration in the serum sample was determined by a calibration curve made by analyzing solutions of known CD5 concentration in the range 0.05–99.26 pM and by the derived linear equation. As shown in Table 2, the recovery rates were in an acceptable 10% range, indicating that the immunosensor had good accuracy in the serum matrix. The fact that a higher concentration of CD5 was obtained than spiked can be explained by the very low amount of CD5 in the serum itself, as well as by the nonspecific sorption of serum matrix components that produces a nonspecific response. Both cause an additional signal independent of the amount of CD5 spiked.

**Table 2:** Detection of CD5 in Human Serum Samples^a^

Added concentration, pM	Detected concentration (*n* = 3), pM	Recovery, %
1.04	1.14 ± 0.16	109.62
26.02	27.93 ± 2.03	107.34
63.53	67.27 ± 3.53	105.89

a *n*, number of measurements.

## 4 Conclusions

This study proposed a signal amplification strategy for the SPR immunosensor based on the use of antibody-functionalized magnetoplasmonic nanoparticles and an indirect sandwich immunoassay format. Due to the high mass and the electromagnetic enhancement in the evanescent field at the SPR sensor surface caused by the localized surface plasmons excited in the nanoparticles, the binding of the mAuNPs–anti-CD5_2B_ to the anti-CD5_2A_/CD5 immune complex caused a drastic increase in SPR signal, thus ensuring significant amplification of CD5 detection. Compared to the direct detection format with the LOD of 1.04 nM and the LOQ of 3.47 nM, the use of mAuNPs–anti-CD5_2B_ allowed reduction of the LOD and LOQ to 8.31 and 27.70 fM, respectively. The immunosensor also showed satisfactory performance in human blood serum (recovery of 1.04 pM of CD5 was 109.62%). These results indicate that the proposed signal amplification strategy has advantageous properties and offers promising potential to significantly increase the sensitivity of SPR immunosensors. This is extremely important for clinical purposes when ultralow concentrations need to be determined. Due to these advantages, it is also likely that the proposed signal amplification strategy based on antibody-functionalized magnetoplasmonic nanoparticles would be useful in the development of other mass-sensitive immunosensors, such as quartz crystal microbalance immunosensors or others.
